# 3-(Ammonio­methyl)­pyridinium bis­(perchlorate)

**DOI:** 10.1107/S1600536813012038

**Published:** 2013-05-11

**Authors:** Imen Bayar, Riadh Kefi, Pedro Sidonio Pereira da Silva, Manuela Ramos Silva, Cherif Ben Nasr

**Affiliations:** a Laboratoire de Chimie des Matériaux, Faculté des Sciences de Bizerte, 7021 Zarzouna, Tunisia; b CEMDRX, Physics Department, University of Coimbra, P-3004-516 Coimbra, Portugal

## Abstract

In the title molecular salt, C_6_H_10_N_2_^2+^·2ClO_4_^−^, the Cl—O bond lengths [anion 1: 1.369 (3)–1.415 (3); anion 2: 1.420 (2)–1.441 (2) Å] and the O—Cl—O angles [anion 1: 105.4 (2)–111.8 (4); anion 2: 107.8 (1)–110.3 (1)°] indicate a slight distortion of the perchlorate anions from regular tetra­hedral symmetry. In the crystal, the components are linked into columns along the *a*-axis direction *via* N—H⋯O and C—H⋯O hydrogen bonds, with stacks of the organic mol­ecules being surrounded by stacks of perchlorate anions.

## Related literature

For general background to perchlorate salts with organic cations, see: Czarnecki *et al.* (1994 ▶); Czupinski *et al.* (2002 ▶, 2006 ▶). For related structures, see: Kapplinger & Keutel (1999 ▶); Ye *et al.* (2002 ▶)
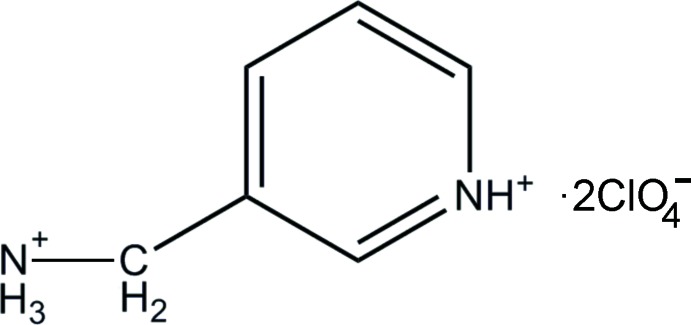


## Experimental

### 

#### Crystal data

C_6_H_10_N_2_^2+^·2ClO_4_^−^
*M* *_r_* = 309.06
Monoclinic, 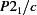

*a* = 5.1947 (1) Å
*b* = 12.1221 (3) Å
*c* = 18.2724 (5) Å
β = 98.067 (1)°
*V* = 1139.24 (5) Å^3^
*Z* = 4
Mo *K*α radiation
μ = 0.61 mm^−1^
*T* = 293 K
0.44 × 0.33 × 0.22 mm

#### Data collection

Bruker APEXII CCD area-detector diffractometer
Absorption correction: multi-scan (*SADABS*; Sheldrick, 2003 ▶) *T*_min_ = 0.765, *T*_max_ = 0.875
26338 measured reflections
3509 independent reflections
3007 reflections with *I* > 2σ(*I*)
*R*_int_ = 0.032

#### Refinement

*R*[*F* ^2^ > 2σ(*F* ^2^)] = 0.051
*wR*(*F* ^2^) = 0.148
*S* = 1.06
3509 reflections
163 parameters
H-atom parameters constrained
Δρ_max_ = 0.70 e Å^−3^
Δρ_min_ = −0.75 e Å^−3^

### 

Data collection: *APEX2* (Bruker, 2003 ▶); cell refinement: *SAINT* (Bruker, 2003 ▶); data reduction: *SAINT*; program(s) used to solve structure: *SHELXS97* (Sheldrick, 2008 ▶); program(s) used to refine structure: *SHELXL97* (Sheldrick, 2008 ▶); molecular graphics: *PLATON* (Spek, 2009 ▶); software used to prepare material for publication: *SHELXL97*.

## Supplementary Material

Crystal structure: contains datablock(s) global, I. DOI: 10.1107/S1600536813012038/lr2104sup1.cif

Structure factors: contains datablock(s) I. DOI: 10.1107/S1600536813012038/lr2104Isup2.hkl

Supplementary material file. DOI: 10.1107/S1600536813012038/lr2104Isup3.cml

Additional supplementary materials:  crystallographic information; 3D view; checkCIF report

## Figures and Tables

**Table 1:** Hydrogen-bond geometry (Å, °)

*D*—H⋯*A*	*D*—H	H⋯*A*	*D*⋯*A*	*D*—H⋯*A*
N1—H1⋯O5	0.86	2.16	2.901 (3)	144
N1—H1⋯O7^i^	0.86	2.36	2.945 (3)	125
N2—H2*A*⋯O2^ii^	0.89	2.09	2.866 (3)	146
N2—H2*A*⋯O6^iii^	0.89	2.54	3.166 (3)	128
N2—H2*B*⋯O6^ii^	0.89	2.10	2.925 (3)	155
N2—H2*B*⋯O3^iv^	0.89	2.47	2.922 (4)	112
N2—H2*C*⋯O1^v^	0.89	2.08	2.933 (3)	160
C5—H5⋯O1^vi^	0.93	2.54	3.350 (4)	145
C6—H6*A*⋯O8^vii^	0.97	2.50	3.137 (3)	123

Symmetry codes: (i) 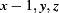; (ii) 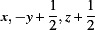; (iii) 
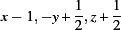
; (iv) 
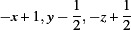
; (v) 
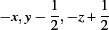
; (vi) 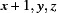; (vii) 
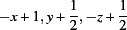
.

**Fig. 1.**
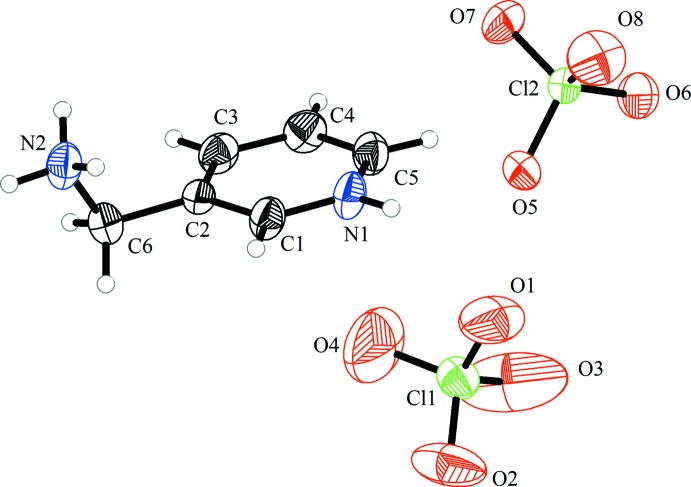
A view of the title compound, showing 50% probability displacement ellipsoids and arbitrary spheres for the H atoms.

**Fig. 2.**
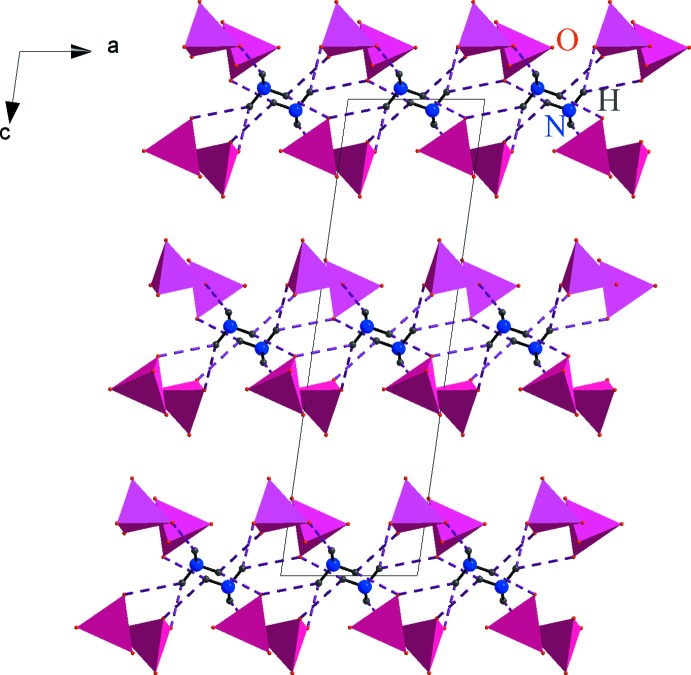
Partial packing of the title compound, viewed down the b axis, showing columns formed between perchlorate anions and ammonium groups.

**Fig. 3.**
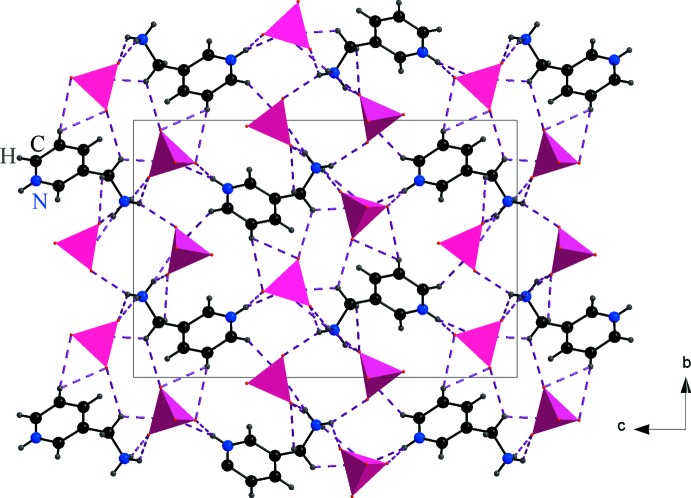
The crystal packing of the title compound viewed along the a axis.

## supplementary crystallographic information

### Comment

Studies of perchlorate salts containing organic cations have had a great deal of attention in recent years, because of their ferroelectric and dielectric behaviour (Czupinski *et al.*, 2002; Czupinski *et al.*, 2006). It was shown that dynamics of pyridinium cations contributes mainly to the mecanism of solid-solid phase transition and leads ferroelectricity in these molecular-ionic crystals (Czarnecki *et al.*, 1994) Here, we report the synthesis and the crystal structure of the title compound (I), (C_6_H_10_N_2_)(ClO_4_)_2_.

The structure consists of one 3-ammoniomethylpyrinidinium dication and two perchlorate anions (Fig. 1). In the crystal, the three H atoms of the ammonium group are involved into N—H···Cl and N—H···O hydrogen bonds: N2—H2C···Cl1(v), N2—H2B···Cl2(ii), N2—H2C···O1(v), N2—H2A··· (O6vi, O2iv) and N2—H2B··· (O6iv, O3vii), the two laters are bifurcated (for the symmetry codes, see Table 1). These hydrogen bonds link the ionic units (NH_3_^+^ and [ClO_4_]^-^) to form columns running along the *a*-axis direction (Fig.2) and situated at *y* = *z* = 1/2 (Fig.3). The organic groups are located between these columns *via* two kinds of hydrogen bonds: a bifurcated N—H···O and two C—H···O: N1—H1 (O5, O7viii), C6—H6A···O8(iii) and C5—H5···O1(vi) (Fig. 3, Table 1). No π-π stacking interactions between the organic rings or C—H··· π interactions towards them are observed. It is worth noting that the C—N—C angles of pyridine are very sensitive to protonation: a pyridinium cation always has an expanded angle of the C—N—C in comparison with the parent pydidine. The angle C(5)—N(1)—C(1) [123.4 (2)°] is consistent with the type of pyridinium cation. The hydrogen atom HN(1), which is deprived from its parent, attaches the nitrogen atom. As expected, the [ClO_4_]^-^ anion has typical tetrahedral geometry where the Cl—O bond lengths and O—Cl—O angles are not equal to one another but vary with the environment around the O atoms. In the title compound, the Cl—O bond lengths vary from 1.369 (3) to 1.415 (3) Å for [Cl(1)O_4_]^-^ anion and from 1.420 (2) to 1.440 (2) Å for [Cl(2)O_4_]^-^ anion are comparable to that previuosly reported for the perchlorate anions (Kapplinger & Keutel, 1999). The O—Cl—O angles range from 105.4 (2) to 111.8 (4)° for the first anion and from 109.04 (15) to 110.33 (13)° for the second one. These values, which are characteristic of perchlorate anions (Ye *et al.*, 2002), clearly indicate that the coordination geometry of the Cl(2) atom can be regarded as being a less distorted tetrahedron than the one of the Cl(1). However, for the Cl(2)O_4_ tetrahedron, all the oxygen atoms are involved in hydrogen bonds, while only three oxygen atoms act as acceptors of hydrogen bonds for the [Cl(1)O_4_] tetrahedron.

### Experimental

3-ammoniomethylpyrinidinium (1 mmol, 0.108 g) was dissolved in a mixture of distilled water (10 ml) and perchloric acid (0.5 ml).The resultant solution was evaporated at room temperature. Crystals of the title compound, which remained stable under normal conditions of temperature and humidity, were isolated after several days and subjected to X-ray diffraction analysis (yield 64%).

### Refinement

All the H-atoms were located in difference Fourier synthesis maps and refined as riding on their parent atoms, using *SHELXL97*(Sheldrick, 2008) defaults.

### Crystal data

**Table:**

C_6_H_10_N_2_^2+^·2ClO_4_^−^	*F*(000) = 632
*M**_r_* = 309.06	*D*_x_ = 1.802 Mg m^−^^3^
Monoclinic, *P*2_1_/*c*	Mo *K*α radiation, λ = 0.71073 Å
Hall symbol: -P 2ybc	Cell parameters from 5314 reflections
*a* = 5.1947 (1) Å	θ = 2.3–30.6°
*b* = 12.1221 (3) Å	µ = 0.61 mm^−^^1^
*c* = 18.2724 (5) Å	*T* = 293 K
β = 98.067 (1)°	Block, colourless
*V* = 1139.24 (5) Å^3^	0.44 × 0.33 × 0.22 mm
*Z* = 4	

### Data collection

**Table:**

Bruker APEXII CCD area-detector diffractometer	3509 independent reflections
Radiation source: fine-focus sealed tube	3007 reflections with *I* > 2σ(*I*)
Graphite monochromator	*R*_int_ = 0.032
φ and ω scans	θ_max_ = 30.6°, θ_min_ = 2.0°
Absorption correction: multi-scan (*SADABS*; Sheldrick, 2003)	*h* = −7→7
*T*_min_ = 0.765, *T*_max_ = 0.875	*k* = −17→17
26338 measured reflections	*l* = −26→26

### Refinement

**Table:**

Refinement on *F*^2^	Primary atom site location: structure-invariant direct methods
Least-squares matrix: full	Secondary atom site location: difference Fourier map
*R*[*F*^2^ > 2σ(*F*^2^)] = 0.051	Hydrogen site location: inferred from neighbouring sites
*wR*(*F*^2^) = 0.148	H-atom parameters constrained
*S* = 1.06	*w* = 1/[σ^2^(*F*_o_^2^) + (0.0782*P*)^2^ + 0.9452*P*] where *P* = (*F*_o_^2^ + 2*F*_c_^2^)/3
3509 reflections	(Δ/σ)_max_ < 0.001
163 parameters	Δρ_max_ = 0.70 e Å^−^^3^
0 restraints	Δρ_min_ = −0.75 e Å^−^^3^

### Special details

**Table:**

Geometry. All e.s.d.'s (except the e.s.d. in the dihedral angle between two l.s. planes) are estimated using the full covariance matrix. The cell e.s.d.'s are taken into account individually in the estimation of e.s.d.'s in distances, angles and torsion angles; correlations between e.s.d.'s in cell parameters are only used when they are defined by crystal symmetry. An approximate (isotropic) treatment of cell e.s.d.'s is used for estimating e.s.d.'s involving l.s. planes.
Refinement. Refinement of *F*^2^ against ALL reflections. The weighted *R*-factor *wR* and goodness of fit *S* are based on *F*^2^, conventional *R*-factors *R* are based on *F*, with *F* set to zero for negative *F*^2^. The threshold expression of *F*^2^ > σ(*F*^2^) is used only for calculating *R*-factors(gt) *etc*. and is not relevant to the choice of reflections for refinement. *R*-factors based on *F*^2^ are statistically about twice as large as those based on *F*, and *R*- factors based on ALL data will be even larger.

### Fractional atomic coordinates and isotropic or equivalent isotropic displacement parameters (Å^2^)

**Table:**

	*x*	*y*	*z*	*U*_iso_*/*U*_eq_	
Cl1	0.11942 (12)	0.50919 (5)	0.12761 (3)	0.04193 (16)	
Cl2	0.82533 (9)	0.14688 (4)	0.09563 (2)	0.03098 (14)	
O1	−0.1534 (5)	0.4964 (2)	0.1114 (2)	0.0912 (10)	
O2	0.2513 (6)	0.4146 (3)	0.10993 (17)	0.0916 (10)	
O3	0.1869 (7)	0.5993 (4)	0.0893 (4)	0.172 (3)	
O4	0.1865 (11)	0.5226 (5)	0.2043 (2)	0.169 (2)	
O5	0.5608 (3)	0.16338 (19)	0.10859 (10)	0.0499 (5)	
O6	0.8768 (4)	0.22306 (18)	0.03930 (11)	0.0523 (5)	
O7	0.9987 (4)	0.16608 (18)	0.16220 (10)	0.0530 (5)	
O8	0.8562 (5)	0.03757 (18)	0.07037 (15)	0.0673 (6)	
N1	0.4671 (4)	0.26538 (17)	0.24604 (10)	0.0400 (4)	
H1	0.4241	0.2266	0.2068	0.048*	
N2	0.3730 (4)	0.18501 (18)	0.47673 (11)	0.0429 (5)	
H2A	0.2868	0.1768	0.5152	0.064*	
H2B	0.5421	0.1917	0.4928	0.064*	
H2C	0.3466	0.1263	0.4474	0.064*	
C1	0.3473 (5)	0.24549 (19)	0.30466 (12)	0.0377 (4)	
H1A	0.2182	0.1919	0.3022	0.045*	
C2	0.4155 (4)	0.30457 (18)	0.36882 (10)	0.0318 (4)	
C3	0.6048 (5)	0.38511 (19)	0.36950 (12)	0.0385 (4)	
H3	0.6529	0.4269	0.4119	0.046*	
C4	0.7224 (5)	0.4036 (2)	0.30740 (14)	0.0417 (5)	
H4	0.8501	0.4575	0.3079	0.050*	
C5	0.6497 (5)	0.3420 (2)	0.24510 (12)	0.0392 (5)	
H5	0.7267	0.3537	0.2028	0.047*	
C6	0.2791 (5)	0.2852 (2)	0.43480 (13)	0.0443 (5)	
H6A	0.3051	0.3487	0.4673	0.053*	
H6B	0.0939	0.2781	0.4185	0.053*	

### Atomic displacement parameters (Å^2^)

**Table:**

	*U*^11^	*U*^22^	*U*^33^	*U*^12^	*U*^13^	*U*^23^
Cl1	0.0443 (3)	0.0361 (3)	0.0473 (3)	0.0052 (2)	0.0129 (2)	0.0049 (2)
Cl2	0.0309 (2)	0.0361 (3)	0.0261 (2)	0.00110 (17)	0.00438 (16)	−0.00042 (15)
O1	0.0420 (11)	0.0839 (18)	0.152 (3)	0.0086 (11)	0.0271 (14)	0.0586 (18)
O2	0.0762 (17)	0.104 (2)	0.0872 (19)	0.0430 (16)	−0.0138 (14)	−0.0469 (16)
O3	0.078 (2)	0.132 (3)	0.295 (6)	−0.031 (2)	−0.009 (3)	0.148 (4)
O4	0.190 (5)	0.245 (6)	0.066 (2)	0.104 (4)	−0.003 (2)	−0.048 (3)
O5	0.0325 (8)	0.0816 (14)	0.0368 (8)	0.0027 (8)	0.0089 (6)	−0.0020 (8)
O6	0.0490 (10)	0.0643 (12)	0.0455 (9)	−0.0003 (9)	0.0130 (8)	0.0202 (9)
O7	0.0437 (9)	0.0765 (13)	0.0356 (8)	−0.0043 (9)	−0.0061 (7)	−0.0026 (8)
O8	0.0796 (16)	0.0426 (11)	0.0782 (15)	0.0089 (10)	0.0055 (12)	−0.0173 (10)
N1	0.0520 (11)	0.0437 (10)	0.0241 (7)	−0.0097 (8)	0.0048 (7)	−0.0045 (7)
N2	0.0466 (11)	0.0530 (12)	0.0296 (8)	−0.0131 (9)	0.0066 (7)	0.0008 (8)
C1	0.0407 (11)	0.0434 (11)	0.0284 (9)	−0.0099 (9)	0.0028 (8)	−0.0009 (8)
C2	0.0318 (9)	0.0379 (10)	0.0256 (8)	0.0052 (7)	0.0037 (7)	−0.0005 (7)
C3	0.0416 (11)	0.0383 (10)	0.0337 (10)	−0.0002 (9)	−0.0009 (8)	−0.0066 (8)
C4	0.0400 (11)	0.0409 (11)	0.0430 (11)	−0.0087 (9)	0.0016 (9)	0.0012 (9)
C5	0.0428 (11)	0.0441 (12)	0.0318 (9)	−0.0024 (9)	0.0089 (8)	0.0049 (8)
C6	0.0426 (12)	0.0597 (14)	0.0325 (10)	0.0077 (10)	0.0116 (9)	0.0003 (10)

### Geometric parameters (Å, º)

**Table:**

Cl1—O3	1.369 (3)	N2—H2B	0.8900
Cl1—O2	1.397 (2)	N2—H2C	0.8900
Cl1—O4	1.405 (4)	C1—C2	1.377 (3)
Cl1—O1	1.415 (3)	C1—H1A	0.9300
Cl2—O8	1.420 (2)	C2—C3	1.385 (3)
Cl2—O7	1.4272 (18)	C2—C6	1.500 (3)
Cl2—O6	1.4357 (18)	C3—C4	1.380 (3)
Cl2—O5	1.4406 (18)	C3—H3	0.9300
N1—C5	1.329 (3)	C4—C5	1.369 (3)
N1—C1	1.334 (3)	C4—H4	0.9300
N1—H1	0.8600	C5—H5	0.9300
N2—C6	1.482 (3)	C6—H6A	0.9700
N2—H2A	0.8900	C6—H6B	0.9700
			
O3—Cl1—O2	111.6 (3)	N1—C1—C2	119.7 (2)
O3—Cl1—O4	111.8 (4)	N1—C1—H1A	120.1
O2—Cl1—O4	105.4 (2)	C2—C1—H1A	120.1
O3—Cl1—O1	107.49 (19)	C1—C2—C3	118.16 (19)
O2—Cl1—O1	111.8 (2)	C1—C2—C6	120.6 (2)
O4—Cl1—O1	108.8 (3)	C3—C2—C6	121.2 (2)
O8—Cl2—O7	110.04 (14)	C4—C3—C2	120.2 (2)
O8—Cl2—O6	109.04 (15)	C4—C3—H3	119.9
O7—Cl2—O6	110.33 (13)	C2—C3—H3	119.9
O8—Cl2—O5	109.69 (14)	C5—C4—C3	119.5 (2)
O7—Cl2—O5	109.86 (11)	C5—C4—H4	120.2
O6—Cl2—O5	107.84 (12)	C3—C4—H4	120.2
C5—N1—C1	123.42 (19)	N1—C5—C4	119.0 (2)
C5—N1—H1	118.3	N1—C5—H5	120.5
C1—N1—H1	118.3	C4—C5—H5	120.5
C6—N2—H2A	109.5	N2—C6—C2	112.65 (19)
C6—N2—H2B	109.5	N2—C6—H6A	109.1
H2A—N2—H2B	109.5	C2—C6—H6A	109.1
C6—N2—H2C	109.5	N2—C6—H6B	109.1
H2A—N2—H2C	109.5	C2—C6—H6B	109.1
H2B—N2—H2C	109.5	H6A—C6—H6B	107.8
			
C5—N1—C1—C2	−1.3 (4)	C2—C3—C4—C5	0.3 (4)
N1—C1—C2—C3	1.3 (3)	C1—N1—C5—C4	0.7 (4)
N1—C1—C2—C6	178.6 (2)	C3—C4—C5—N1	−0.2 (4)
C1—C2—C3—C4	−0.8 (3)	C1—C2—C6—N2	78.7 (3)
C6—C2—C3—C4	−178.1 (2)	C3—C2—C6—N2	−104.1 (3)

### Hydrogen-bond geometry (Å, º)

**Table:**

*D*—H···*A*	*D*—H	H···*A*	*D*···*A*	*D*—H···*A*
N1—H1···O5	0.86	2.16	2.901 (3)	144
N1—H1···O7^i^	0.86	2.36	2.945 (3)	125
N2—H2*A*···O2^ii^	0.89	2.09	2.866 (3)	146
N2—H2*A*···O6^iii^	0.89	2.54	3.166 (3)	128
N2—H2*B*···O6^ii^	0.89	2.10	2.925 (3)	155
N2—H2*B*···O3^iv^	0.89	2.47	2.922 (4)	112
N2—H2*B*···Cl2^ii^	0.89	2.96	3.600 (2)	131
N2—H2*C*···O1^v^	0.89	2.08	2.933 (3)	160
N2—H2*C*···Cl1^v^	0.89	2.97	3.654 (2)	135
C5—H5···O1^vi^	0.93	2.54	3.350 (4)	145
C6—H6*A*···O8^vii^	0.97	2.50	3.137 (3)	123

Symmetry codes: (i) *x*−1, *y*, *z*; (ii) *x*, −*y*+1/2, *z*+1/2; (iii) *x*−1, −*y*+1/2, *z*+1/2; (iv) −*x*+1, *y*−1/2, −*z*+1/2; (v) −*x*, *y*−1/2, −*z*+1/2; (vi) *x*+1, *y*, *z*; (vii) −*x*+1, *y*+1/2, −*z*+1/2.
